# Enhancing neuroprosthesis calibration: the advantage of integrating prior training over exclusive use of new data

**DOI:** 10.1088/1741-2552/ad94a7

**Published:** 2024-11-29

**Authors:** Caleb J Thomson, Troy N Tully, Eric S Stone, Christian B Morrell, Erik J Scheme, David J Warren, Douglas T Hutchinson, Gregory A Clark, Jacob A George

**Affiliations:** 1 Department of Biomedical Engineering, University of Utah, Salt Lake City, UT 84112, United States of America; 2 Institute of Biomedical Engineering, University of New Brunswick, Fredericton, New Brunswick E3B 5A3, Canada; 3 Department of Electrical and Computer Engineering, University of Utah, Salt Lake City, UT 84112, United States of America; 4 Department of Orthopaedics, University of Utah, Salt Lake City, UT 84112, United States of America; 5 Department of Mechanical Engineering, University of Utah, Salt Lake City, UT 84112, United States of America; 6 Department of Physical Medicine and Rehabilitation, University of Utah, Salt Lake City, UT 84112, United States of America

**Keywords:** neuroprosthesis, brain–computer interface, EMG, myoelectric, prosthetic, dataset aggregation

## Abstract

*Objective.* Neuroprostheses typically operate under supervised learning, in which a machine-learning algorithm is trained to correlate neural or myoelectric activity with an individual’s motor intent. Due to the stochastic nature of neuromyoelectric signals, algorithm performance decays over time. This decay is accelerated when attempting to regress proportional control of multiple joints in parallel, compared with the more typical classification-based pattern recognition control. To overcome this degradation, neuroprostheses and commercial myoelectric prostheses are often recalibrated and retrained frequently so that only the most recent, up-to-date data influences the algorithm performance. Here, we introduce and validate an alternative training paradigm in which training data from past calibrations is aggregated and reused in future calibrations for regression control. *Approach.* Using a cohort of four transradial amputees implanted with intramuscular electromyographic recording leads, we demonstrate that aggregating prior datasets improves prosthetic regression-based control in offline analyses and an online human-in-the-loop task. In offline analyses, we compared the performance of a convolutional neural network (CNN) and a modified Kalman filter (MKF) to simultaneously regress the kinematics of an eight-degree-of-freedom prosthesis. Both algorithms were trained under the traditional paradigm using a single dataset, as well as under the new paradigm using aggregated datasets from the past five or ten trainings. *Main results.* Dataset aggregation reduced the root-mean-squared error (RMSE) of algorithm estimates for both the CNN and MKF, although the CNN saw a greater reduction in error. Further offline analyses revealed that dataset aggregation improved CNN robustness when reusing the same algorithm on subsequent test days, as indicated by a smaller increase in RMSE per day. Finally, data from an online virtual-target-touching task with one amputee showed significantly better real-time prosthetic control when using aggregated training data from just two prior datasets. *Significance.* Altogether, these results demonstrate that training data from past calibrations should not be discarded but, rather, should be reused in an aggregated training dataset such that the increased amount and diversity of data improve algorithm performance. More broadly, this work supports a paradigm shift for the field of neuroprostheses away from daily data recalibration for linear classification models and towards daily data aggregation for non-linear regression models.

## 1. Introduction

Neuroprostheses—prostheses directly integrated with an individual’s neuromuscular system—have the potential to enable dexterous control of multiarticulate bionic arms. Neuroprostheses typically operate under a supervised machine-learning approach, where an algorithm is first trained to correlate neuromyoelectric activity with an individual’s motor intent. After the algorithm is trained, the algorithm can then be used to predict the kinematics of a multiarticulate bionic arm based on neuromyoelectric signals in real time.

One of the most fundamental ways to improve the accuracy of a machine-learning algorithm is to increase the amount of training data [1–3]. However, collecting more training data in a given session is often not feasible with neuroprostheses. During algorithm training for a neuroprosthesis, the user must actively perform labelled kinematic movements while their neuromuscular activity is recorded synchronously [4–10]. Requiring a longer duration of active participation from the user is difficult due to limited user time and both mental and physical fatigue [11–15]. As such, the vast majority of neuroprostheses utilize only a brief training session (<30 min) that is collected immediately before use [4, 6, 7, 9, 10]. Similarly, commercially available non-invasive myoelectric control algorithms, like the Complete Control pattern recognition system (Coapt, LLC), also utilize a short training session before use [16]. This training session is also commonly referred to as a training calibration or simply a calibration. Most training sessions for neuroprostheses take 30 min or less. For example, recent works have utilized 20 min [8], five to 10 min [4, 10], or even as little as 50 s for two degrees of freedom [17]. Short training sessions are important to reduce the burden on the patients using the neuroprostheses.

Ideally, a short training session would provide accurate and robust prosthetic control for an extended period of use. However, due to temporal variations in signal quality [18–21] and signal location [22–24], algorithm performance decays over time [25–28]. Using implanted neuromyoelectric devices improves signal quality over surface signals [29–31], but even implanted neural [29, 32–36] and myoelectric [8, 29, 37–40] signals change over time, as can the control strategies used by users. Thus, the current paradigm typically requires daily or task-specific recalibration, in which a short training session is performed at the start of the day [41–45] or before a specific task [9, 46]. Even commercially available non-invasive myoelectric control algorithms are often recalibrated multiple times per day [47–49].

The current paradigm results in the user performing dozens to hundreds of short training datasets each year [32, 37, 41]. At the start of each day or each task, the user performs another short training session, the new data are used to retrain the algorithm, and the old data are discarded. Recalibration or retraining of myoelectric control algorithms can be time-consuming and burdensome for the user [50]. One potential solution would be to aggregate computer-generated data with the original training data. This is commonly called data augmentation, sometimes called data aggregation [51]. Data augmentation has been used to improve deep learning control algorithms [51], help algorithm robustness to electrode shift with surface EMG (sEMG) [52, 53], and improve between-day algorithm robustness [51, 54]. Another solution specifically used with sEMG is aggregating data from multiple participants to generalize algorithms across users, commonly called transfer learning [55, 56]. With enough data, transfer learning approaches can reduce the need for per-person calibration [55], while others still require a short per-person calibration [56].

Importantly, with the exception of [51], the vast majority of these techniques have been shown with discrete classification of gestures. These classification algorithms use large time windows of EMG data that often encompass the entire duration of a gesture [57, 58]. Intraclass variability is less of a concern when there is a large separation among classes. In contrast, here we explore a more complex and novel challenge of simultaneous and proportional control of multiple degrees of freedom in real time. Under this approach, EMG data is used to regress the precise kinematics of each joint with millisecond temporal resolution. Regression-based control is more akin to endogenous control of the human hand [59] and has been shown to outperform classification-based control in functional tasks [60]. Regression is distinct from classification in that the magnitude of the EMG provides crucial information for the proportional kinematic prediction. As such, regression is highly sensitive to signal changes and recalibration frequency is particularly high.

Here, we propose an alternative paradigm for regression algorithms suitable for proportional control: rather than replace old data with new data or augment it with computer-generated data, we simply propose to aggregate old data with new data. Thus, when the algorithm is retrained at the start of each day or task, the algorithm sees *both* the new data and the old data from *all* prior training sessions. The result is an increasingly large dataset with increasingly more variability (due to the aforementioned temporal variations). This new paradigm raises a key question: does including old–potentially outdated and invalid data–degrade overall algorithm performance, or does the additional data and variability increase algorithm accuracy and robustness?

To answer this question, we first utilized existing training datasets from three transradial amputees with implanted intramuscular electromyographic recording leads (iEMGs). We found that including past datasets improved algorithm accuracy and that improvements were more pronounced for a non-linear algorithm than a linear one. Furthermore, we found that including past datasets also made the algorithm more robust, as indicated by slower algorithm degradation over time. Finally, we explored this approach in real time using a fourth transradial amputee with implanted iEMGs. For this participant, we found that real-time prosthetic control was significantly better when aggregating old and new data instead of training exclusively on new data collected immediately before the task. These results demonstrate that aggregating, rather than discarding, past datasets can improve prosthetic control. Furthermore, in the case of iEMG, significant improvements can be seen after aggregating just two prior datasets. Thus, this manuscript supports a paradigm shift for the field of neuroprostheses away from daily data recalibration for linear algorithms and towards daily data aggregation for non-linear algorithms.

## 2. Methods

### 2.1. Participants

Four participants with transradial amputation participated in this study. Three have been described previously as subjects: S5, S6 and S7. The first participant (S5) was a 43-year-old male with bilateral transradial amputations that occurred 24 years prior due to trauma [61, 62]. The next subject (S6) was a 57-year-old male with left transtibial and left transradial amputations that occurred 13 yr prior due to trauma [61, 63, 64]. The third subject (S7) was a 48-year-old male with a right transradial amputation that was undertaken electively due to chronic complex regional pain syndrome [41, 65]. The fourth subject (S8) has not been reported previously. S8 was a 57 year-old female with a right transradial amputation that occurred 1.5 yr prior due to trauma. All amputee participants were evaluated by a physician and a psychologist for their willingness and ability to participate in the study. All surgeries and experiments were performed with informed consent from the participants and following protocols by the University of Utah Institutional Review Board and the Department of Navy Human Resources Protection Program. S8 was also covered by an approved FDA Investigational Device Exemption Early Feasibility Study protocol, which had not been required for previous participants.

### 2.2. Implanted devices

All participants received iEMGs and two to three Utah Slanted Electrode Arrays (USEAs; Blackrock Neurotech, Salt Lake City, UT, USA) implanted in residual arm nerves. The present report focuses on iEMG recordings.

Participants S5, S6 and S7 were implanted with eight iEMGs (Ripple Neuro LLC, Salt Lake City, UT, USA) with four contacts each for a total of 32 contacts [41, 61, 63] (table 1). Each lead was intended to target a different forearm extensor or flexor muscle. A separate iEMG lead with two contacts was implanted proximal and posterior to the elbow to provide an electrical reference and ground. The iEMG electrodes exited the arm through a percutaneous incision and were mated with associated Front Ends (Ripple Neuro LLC) for filtering and amplifying EMG signals.

**Table 1.** Transradial amputee participants.

Subject	# of iEMG contacts	Time with iEMGs (days)	# of total iEMG training datasets	Days spanned by datasets	Analysis
S5	32	84	16	84	Offline
S6	32	425	25	147	Offline
S7	32	503	25	315	Offline
S8	20	161	3	54	Online

iEMGs were surgically removed after 84 d for S5 due to an infection at the percutaneous wire passage site. S5 recovered fully after device extraction and antibiotic treatment. iEMGs were surgically removed at 425 and 503 d for participants S6 and S7, respectively, due to prior mutual agreements regarding study duration between the volunteer participants and the experimenters.

**Figure 1.**
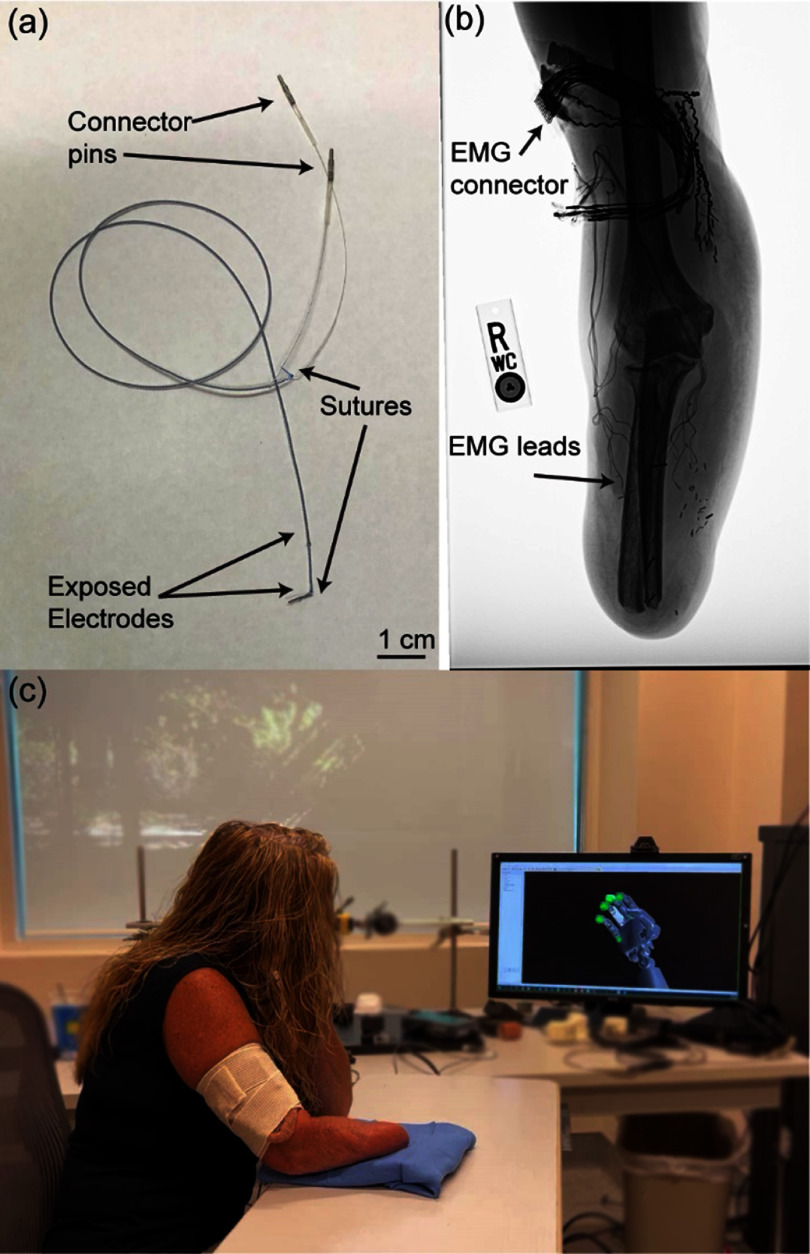
Experimental setup. (a) Data were collected from intramuscular electromyographic (iEMG) recording leads [37, 66]. (b) iEMGs were implanted into the residual extrinsic forearm muscle of four participants, S5–S8. (c) iEMGs were used to complete an online target-touching task [41, 65] with a virtual bionic arm in real time. Images all pertain to S8. Utah Slanted Electrode Arrays (USEAs) were also implanted in each patient but were irrelevant to this study.

Participant S8 similarly had iEMGs implanted into the residual limb (figures 1(a) and (b)). Due to FDA regulations, the iEMGs used for S8 differed slightly from those used with previous participants. Participant S8 was implanted with ten custom bipolar iEMG leads (Synapse Biomedical, Oberlin, OH, USA) [37, 66] with two contacts each for a total of 20 contacts. Six leads targeted the flexors, and four leads targeted the extensors. Two separate iEMG leads were implanted proximal and posterior to the elbow to provide an electrical reference and ground. The iEMG leads exited the arm through a percutaneous incision and were wired into a Samtec connector to mate with the associated Front Ends (Ripple Neuro LLC) for filtering and amplifying signals.

S8 elected to have all iEMG and USEA devices surgically removed after 161 d due to chronic inflammation, generalized discomfort, and slightly infected skin portals. Infections subsequently worsened but resolved fully after antibiotic treatment.

### 2.3. Data collection

Participants were involved in research on the control of advanced, multiarticulate prosthetic devices. During daily experiments of prosthetic control, training datasets were collected to allow the user to control a virtual bionic arm or physical prosthesis [4, 41, 61, 63, 65, 67]. The datasets consisted of prerecorded movements of a 12-degree-of-freedom (DOF) virtual bionic arm (MSMS; John Hopkins Applied Physics Lab, Baltimore MD, USA; figure 1(c)) or a 6-DOF physical prosthesis (‘LUKE’ Arm; DEKA, Manchester, NH, USA). The 12 DOFs of the virtual bionic arm are flexion and extension of the fingers (D1 through D5) and wrist, abduction and adduction of D1, D2, D4, and D5, and pronation, supination, and radial and ulnar deviation of the wrist. The 6 DOFs of the physical prosthesis are flexion and extension of the fingers and wrist (with D3, D4, and D5 coupled), abduction and adduction of the D1, and pronation and supination of the wrist. EMG was recorded with iEMG leads while the participants attempted to mimic the prerecorded movements with their phantom limb, moving their phantom fingers and contracting their muscles in proportion to the virtual fingers. In general, datasets consisted of participants mimicking individual movements of flexion and extension of the fingers and wrist, intrinsic thumb movement, and pronation and supination of the wrist. Additionally, finger movements were sometimes combined to mimic the movement of the prosthesis (e.g. combining movements of digits three, four, and five) or to recreate common grasps (e.g. pinching with the D1 and D2 or whole hand grasping). Each movement was repeated four to 20 times, with the most common number of trials being five and ten. The time to collect each training set was between four and 15 min, depending on the number of trials, as well as variable rise and hold times for each movement. Additional details regarding the datasets can be found in table S1. The kinematic position for 12 DOFs was recorded during the movements and was limited between −1 and 1, where −1 corresponded to maximum extension/adduction/supination, +1 corresponded to maximum flexion/abduction/pronation, 0 corresponded to the hand at rest [4]. The kinematic data from the virtual bionic arm or physical prosthesis was combined with the recorded EMG signals during the data collection to make the dataset.

EMG was sampled at 1 kHz using the Grapevine (Nomad or Summit) System (Ripple Neuro, LLC, Salt Lake City, UT, USA). For S5, S6, and S7, there were 32 iEMG contacts, and S8 had 20 iEMG contacts. Each iEMG contact collected one channel of continuous EMG. The EMG channels were band-pass filtered with cutoff frequencies of 15 Hz (sixth-order high-pass Butterworth filter) and 375 Hz (second-order, low-pass Butterworth filter). Notch filters were applied at 60, 120, and 180 Hz. Differential EMG signals were calculated for all possible pairs of channels, resulting in 496 (32 choose 2) differential recordings for S5, S6, and S7, and 190 (20 choose 2) differential recordings for S8, in addition to single-ended recordings. The mean absolute value (MAV) over a sliding 300 ms window was calculated at 30 Hz for all the single-ended channels and differential pairs. The resulting EMG feature set consisted of the 300 ms smoothed MAV on 528 EMG channels (S5–S7) or 210 EMG channels (S8), calculated at 30 Hz. The final training data consisted of kinematic recordings of the 12 DOFs from the predetermined movements of the physical prosthesis or virtual bionic arm and the EMG feature set.

### 2.4. Control algorithm description

Algorithms were trained to estimate and predict the prosthesis kinematics based on the EMG feature set. To account for the participant’s reaction time in mimicking the hand movements, we shifted the recorded kinematics by a lag determined by cross-correlating the kinematics and EMG recordings [65, 68]. The algorithms used here were a modified Kalman filter (MKF) [41], and a convolutional neural network (CNN) [65]. Both control algorithms were implemented in MATLAB 2020b (MathWorks, Natick, MA, USA). A summary of these algorithms follows.

#### 2.4.1. MKF

We used a Kalman filter (KF) defined in previous work [4, 41, 63, 65, 67, 69–71] to estimate and predict motor intent from the continuous EMG signals. Ad-hoc modifications to this KF have been described in our previous work [41], leading to the current MKF. Briefly, the output is modified by a threshold, such that the modified output will remain at zero until the absolute value of the nonmodified output is greater than the threshold. As in previous publications [63, 65], we used a default threshold value of 0.2. The MKF has been used previously for myoelectric control [41, 63, 65, 72], and the detailed mathematical justification, construction, and parameters of the KF have been outlined [69]. The baseline MAV was subtracted from the features before training and testing the KF. We assumed that the EMG features were normally distributed and relied on the KF covariance matrix to inherently address differences among them. The EMG feature set (528 or 210 channels) was reduced to 48 channels using a stepwise Gram–Schmidt channel-selection algorithm [73]. A single KF was used to predict all DOFs of the prosthetic hand. We limited outputs of the KF between −1 and 1, where −1 corresponded to maximum extension/adduction/supination, +1 corresponded to maximum flexion/abduction/pronation, and 0 corresponded to when the hand was at rest [4]. We used 100% of the data in the training datasets to train each MKF.

#### 2.4.2. CNN

The CNN used in this study was previously defined in [65]. This CNN was selected as it has been successful in regression for real-time control of up to 8 DOFs [46, 65, 74]. Other CNN architectures have been used in regression for prosthetic control [51, 75, 76]; however, they are either limited in the number of DOFs controlled [75, 76] or used only in offline analyses [51]. The CNN was trained on a random 60% of the movements in the training datasets and validated on the remaining 40%. Training automatically terminated once the loss of the validation data did not decrease for 20 iterations. This allowed the CNN to train sufficiently, but stopped the training before the CNN could overfit to the training data. The CNN was trained using a Stochastic Gradient Descent with a momentum solver with a learning rate of 0.00001, as in our previous work [77].

The CNN input and structure followed [65]. The CNN input was an *N* × 10 image consisting of *N* features (528 for S5, S6, and S7, and 210 for S8) sampled at the current time and nine previous time points (samples are acquired at 30 Hz). The CNN architecture consisted of a single convolutional layer, two fully connected layers, rectified linear activation function (ReLu) activation between layers, and a regression output. A 1 × 5 kernel was used for the convolution, such that the convolution was only across time and not across the feature set of EMG features. A total of ten convolutional filters were used to produce an *N* × 6 × 10 output feature map. The output of the convolutional layer was then passed through a ReLu activation layer before being passed to the first fully connected layer. The output of the first fully connected layer was also passed through a ReLu activation layer before being passed to the second connected layer. Both fully connected layers consisted of 2 N neurons; thus, the total size of the network depended on the number of input features being used. The output of the second fully connected layer was then fed into a final fully connected layer that produced 12 regression outputs, one for each possible DOF in the bionic hand.

### 2.5. Offline analysis

#### 2.5.1. Dataset aggregation

A subset of prior participants’ datasets (S5–S7) with a consistent format was selected for the offline analysis. The subset of datasets identified all utilized the same 8 DOFs: individual flexion and extension of D1 through D5, abduction and adduction of D1, pronation and supination of the wrist, and flexion and extension of the wrist. Simultaneous flexions and extensions of D1–D5 were also present in the datasets. The total number of datasets available for participants S5, S6, and S7 were 16, 25, and 25, respectively. Preliminary analyses suggested diminishing returns after aggregating more than ten datasets [77]; thus, the first ten datasets of each participant were reserved for training, and the remaining six (for S5, ten trials per movement in each dataset) or 15 (for S6, five or ten trials per movement in each dataset; average 9.3 trials per movement; and S7, four to ten trials per movement in each dataset; average 6.8 trials per movement) were reserved for testing. The exact movements, number of trials, and the duration of the training are listed in table S1.

**Figure 2.**
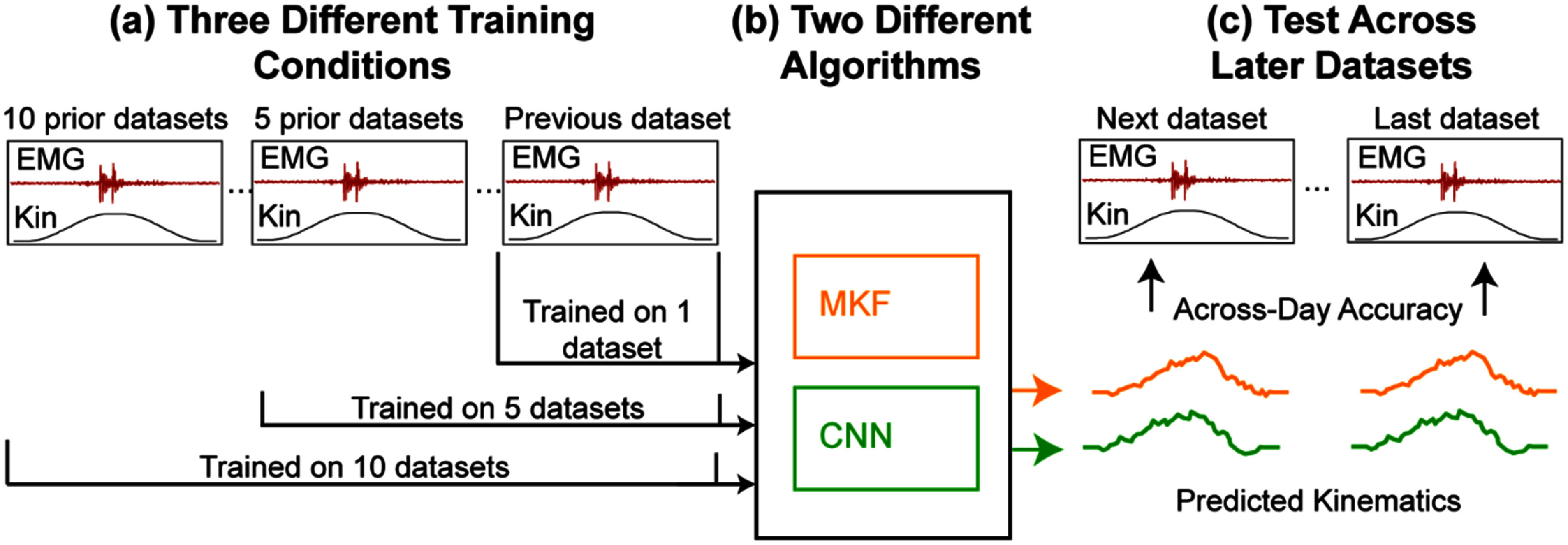
Offline analysis. (a) Prior datasets were aggregated to create three aggregated training datasets using data from the previous one, five, or ten datasets. Training data consisted of EMG recordings (EMG) and the participants desired kinematics (Kin). (b) Two different algorithms (MKF and CNN) were trained on each of the three aggregated training datasets, yielding a total of 6 conditions. (c) The six conditions were then tested on novel, unseen datasets not used by the algorithms in training. Performance metrics included across-day accuracy relative to the recorded kinematics root mean square error (RMSE) of both intended and unintended movements. Next indicates the following dataset after the training datasets and last indicates the latest dataset used for testing.

Three unique aggregated training datasets were generated: (1) aggregating ten datasets prior to the testing datasets (datasets 1–10; totaling 105 trials per movement for S5, 85 for S6, and 49 for S7), (2) aggregating five datasets prior to the testing datasets (datasets 6–10; totaling 60 trials per movement for S5, 40 for S6, and 20 for S7), and (3) using only a single dataset prior to the testing datasets (dataset 10; 10 trials per movement for S5, 5 trials for S6, and 4 for S7). Training datasets were collected between a few days and a few months before the first testing dataset. A MKF and CNN were trained using these three aggregated training datasets and then tested on the novel testing datasets (figure 2).

#### 2.5.2. Metrics

Offline analyses measured control algorithm performance using the root-mean-squared error (RMSE) between algorithm predictions and target kinematics that the participants attempted to mimic. The RMSE was divided into two categories: intended movement RMSE and unintended movement RMSE (i.e. cross-talk) [41, 77]. Intended movement RMSE captures the ability of an individual to precisely control a given degree of freedom and is calculated as the error between the target position and the kinematic for the DOF in which the target is nonzero. Unintended movement RMSE captures the ability of an individual to move a given degree of freedom in isolation and is calculated as the error between the target resting positions and the kinematics for the remaining DOFs in which the target is zero. For example, if the target trajectory was to perform isolated movement of the thumb, then the intended movement would be measured as the difference between the target trajectory of the thumb and the actual kinematic trajectory of the thumb; unintended movement would be measured for all of the other DOFs, as the difference between the actual kinematic trajectories and the target resting positions. Because multiple values of unintended movement are produced per trial, the values are averaged across the DOFs to create a single value unintended movement RMSE. We directly compared the intended movement RMSE and unintended movement RMSE among the two algorithms and three aggregated training datasets (6 conditions total). We also evaluated the algorithm robustness by measuring changes in RMSE for each degree of freedom over the 6 (S5) or 15 (S6 and S7) testing datasets per participant. For each of the 8 degrees of freedom, we calculated a linear line of best fit using least squares for RMSE of the testing datasets as a function of days since training, resulting in 24 slopes (*N* = 3 participants × 8 DOFs).

#### 2.5.3. Feature space analysis

To illustrate differences in feature space, we performed principal component analysis (PCA) across the datasets for each participant, using the single value decomposition approach. PCA is an orthogonal linear transformation that projects multivariate data onto a new coordinate system termed as the principal components. The principal components are ordered where the first principal component explains the largest amount of variance in the original space down to the last principal component, which explains the least [78, 79]. PCA was completed per participant by aggregating the EMG features from each dataset into one matrix for each participant, resulting in one set of principal components for each participant. For visualization, the first two principal components were separated by each movement per participant.

### 2.6. Online activities

#### 2.6.1. Training

As with the prior participants, we instructed S8 to mimic the programmed movements of a virtual bionic hand to correlate EMG activity to intended hand movements. As S8 mimicked the virtual hand movements with their phantom limb, we recorded, in synchrony, the kinematics of the virtual hand and EMG activity. The participant completed a total of three training sessions over the span of three experimental sessions. Each training session consisted of 6 degrees of freedom corresponding to the prosthesis S8 used (‘LUKE’ arm; DEKA, Manchester, NH, USA). The six degrees of freedom were individual flexion and extension of D1, D2, and D3-D5, abduction and adduction of D1, flexion and extension of the wrist, and pronation and supination of the wrist. Each movement was repeated six times before advancing to the next movement. Each movement was 7 s in duration, consisting of a 3-s flexion/extension away from the resting hand position, a 1-s hold-time at the maximum distance away from the resting hand position, and a 3-s extension/flexion returning to the resting hand position. This duration of movements has been shown to improve the ability to predict motor intent using both linear and non-linear algorithms [65]. The cumulative duration, across all movements, for each training session was around 13 min.

#### 2.6.2. Target-touching task (TTT)

As in previous work [41, 65], we used a virtual TTT to quantify user and algorithm performance. This task involves controlling a virtual bionic arm (MSMS; John Hopkins Applied Physics Lab, Baltimore, MD, USA), where the participant is provided real-time visual feedback (figure 1(c)). In this task, the participant actively controlled the virtual MSMS hand and attempted to move select DOFs to a target location and hold the remaining DOFs at rest. Target locations for the selected DOFs were at 50% and 100% of the maximum amount of flexion/extension possible to evaluate proportional control for the selected DOF; the remaining DOFs had targets at rest. The participant was instructed to hold all DOFs within their target location for the trial duration. The participant viewed the target locations and the hand kinematics in real-time on a computer screen. One target was shown for each DOF, and if the corresponding kinematic was within ±15% of the target location, the target would change colour. A red target indicated they were further than ±15% from the target, and a green target indicated they were within ±15% of the target. Each test trial lasted 5 s, with a 2 s wait time between trials. A total of ten trials, five at 50% and five at 100% maximum distance from rest were tested for the 12 different movements (both directions of the 6 degrees of freedom).

The participant completed the TTT three times, once per each of the three experimental sessions. The experimental sessions were about a month apart due to participant scheduling. The participant did not complete the TTT in between the experimental sessions. The first session provided a baseline performance level for the user with a CNN trained on one dataset. In the subsequent two experimental sessions, we compared a CNN trained on that day’s training dataset against a CNN trained on an aggregated training dataset. The single training dataset CNN and the aggregated training dataset CNN were blinded from the participant and appeared in a pseudorandomised, counterbalanced order to eliminate the possibility of any learning effects within a session.

#### 2.6.3. Metrics

For the online analysis, we compared the algorithm performance from the two training datasets on the TTT using five metrics: (1) intended movement RMSE, (2) unintended movement RMSE, (3) percent time in the target 15%-error window, (4) the mean longest continuous-hold duration (i.e. hold duration) within the desired 15%-error window around the target location [65], and (5) the log mean absolute jerk [80, 81]. The percentage time in target determines the overall performance on the task, and the mean longest continuous hold duration extrapolates performance to a more functional metric (such as the ability to hold an object without dropping it). Finally, the log mean absolute jerk measures the relative smoothness of the control, which is important when using a prosthesis in activities of daily living.

Intended and unintended movement RMSE were calculated as the error between the target position(s) and the kinematic(s) on a trial-by-trial basis. To calculate RMSE devoid of the participant’s reaction time, we shifted the recorded kinematics by a lag that was determined by cross-correlating the kinematic estimate and target location signals [68]. Consistent with prior work [41, 65], this alignment was applied across all experimental conditions for a given session so that there would be no bias affecting one experimental condition more than another. Additionally, the RMSE was calculated from the 15%-error window, such that the RMSE was zero if the DOF was anywhere within the 15%-error window (consistent with the visual feedback the participant received). The relative distance between the target window and kinematics appeared to be consistent within a trial since the data were temporally aligned to account for reaction time.

### 2.7. Statistical analysis

All statistical analyses were completed using the Statistics and Machine Learning Toolbox in MATLAB 2021b (MathWorks, Natick, MA, USA).

#### 2.7.1. Offline analyses

The intended and unintended movement RMSE data were determined to be parametric via the Anderson–Darling test (*p* > 0.05), so parametric statistical analyses were performed. The slopes of the change in intended movement RMSE through time were determined to be nonparametric via the Anderson–Darling test (*p* < 0.05), so nonparametric statistical analyses were performed. A three-way analysis of covariance (ANCOVA) was used to assess algorithm accuracy and compare the intended and unintended RMSE for the different participants, algorithms, and number of training datasets. The three factors were participant, algorithm, and number of training datasets, where dataset size was treated as a continuous variable. A one-way nonparametric ANCOVA (Kruskal–Wallis) was used to compare the slope of RMSE over time for the different training datasets to assess algorithm robustness. Following the ANCOVA, subsequent post-hoc paired *t*-tests (Wilcoxon signed rank test) were performed with Tukey’s honestly significant difference criterion correction for multiple comparisons.

Further supplemental analyses were completed to investigate the significant effects of the ANCOVAs, including individual subject analyses. Where significance was found, subsequent pairwise comparisons with correction for multiple comparisons were completed.

#### 2.7.2. Online analyses

The online results of the TTT for each outcome metric were determined to be nonparametric through the Anderson–Darling test (*p* < 0.05); therefore, nonparametric statistical tests were used. To assess baseline user performance and user learning, each of the five outcome metrics was compared across sessions for the CNNs trained on the single training dataset from that day. To assess the benefit of aggregated data, outcome metrics were compared between the CNN trained on the single training dataset from that day and the CNN trained on the aggregated training datasets. Separate one-way non-parametric ANOVAs (Kruskal–Wallis) were performed for each metric. If any significance was found, subsequent pairwise comparisons (Wilcoxon rank sum tests, *N* = 120 trials on session one, and *N* = 240 sessions two and three) were made using Tukey’s honestly significant difference criterion correction for multiple comparisons.

## 3. Results

### 3.1. Aggregating prior datasets improved algorithm accuracy by reducing unintended movements

A common challenge when controlling multiple degrees of freedom with proportionality is the unintended movement of what should be a stationary degree of freedom. For example, when grasping a fragile object, one may intend to flex all five of their digits while keeping their wrist stationary to gently pick up the object. In this example, any movement of the wrist would be unintended and have a negative impact on the task. We first explored the impact of aggregating prior datasets on unintended movements. To quantify this, we calculated the unintended movement RMSE of two control algorithms (MKF and CNN) trained on three different amounts of aggregated training data (one, five, and ten prior datasets).

**Figure 3.**
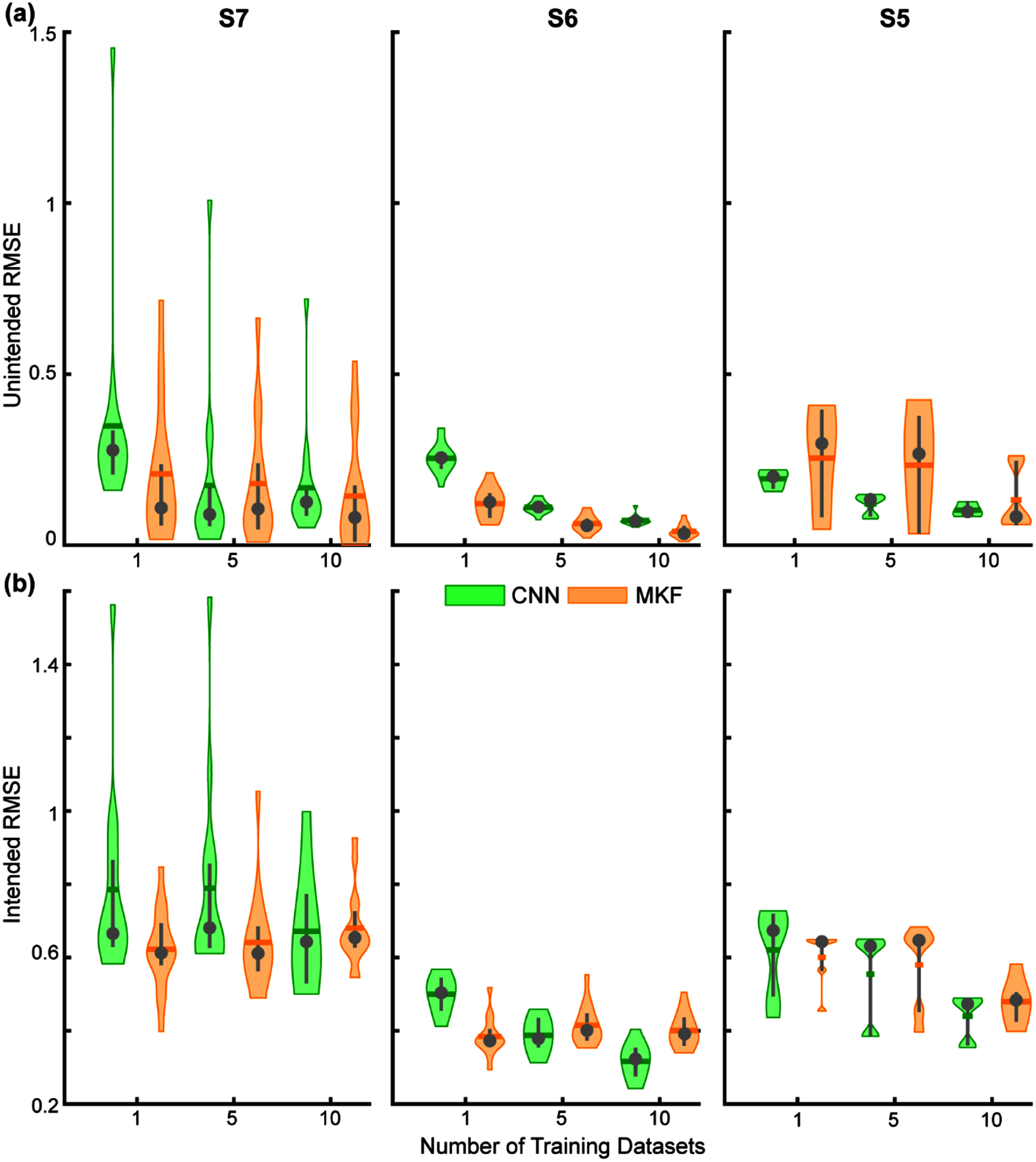
Impact of aggregated training datasets on algorithm performance. (a) Increasing the number of aggregated training datasets reduced the RMSE of unintended movements (*p* < 0.001, ANCOVA). No significant effects were found among the algorithms or participants. (b) Increasing the number of aggregated training datasets also reduced the RMSE of intended movements (*p* < 0.001, ANCOVA). However, this effect was largely associated with the CNN, which outperformed the MKF (*p*< 0.001, ANCOVA), particularly with an increased number of aggregated training datasets (*p* < 0.01, ANCOVA). Collapsed data across participants showed significant improvement in intended RMSE aggregating ten datasets over one with the CNN (*p* < 0.05) and individually for participants S5 (*p* < 0.05) and for S6 (*p* < 0.001) but not for S7. Only participant S6 showed improvement aggregating five datasets over one and ten datasets over five with the CNN (*p* < 0.001). There was no significant improvement in the intended RMSE aggregating more datasets with the MKF for any individual participant or in the collapsed data. Violin plots show the kernel density estimation. Coloured horizontal lines denote the mean, grey circles denote the median, and grey vertical lines denote the interquartile range. *N* = 6 test datasets for S5 and *N* = 15 test datasets for S6 and S7. Boxplot representations in figure S2.

We found that increasing the number of aggregated training datasets significantly reduced unintended movement RMSE (*p* < 0.001, ANCOVA; figure 3(a)). Relative to the traditional paradigm for regression algorithms using a single training dataset, aggregating five datasets resulted in a 40% improvement and aggregating ten datasets resulted in a 54% improvement. No other significant effects were found, suggesting no differences among the algorithms or participants. Subsequent pairwise comparisons among the different amounts of aggregated training data showed a significant reduction in unintended movement when training on five or ten datasets relative to training on one dataset (*p* < 0.01; figure S1(A)). No significant effect was observed between five and ten datasets, although there was a trend towards significance for participant S6 (*p* = 0.057, figure S1(A)).

### 3.2. For the CNN, aggregating prior datasets also improved algorithm accuracy by enhancing accuracy of intended movements

In the above example of grasping a fragile object, the user would be actively attempting to modulate the position of the digits, with proportionality, such that the force exerted by the prosthesis is greater than the force needed to pick up the object against gravity and less than the force needed to break the object. When regressing kinematic position in real-time, errors in the intended movement of active degree(s) of freedom would negatively impact task performance by causing the user to break or drop the object. To this end, we next explored the impact of aggregating prior datasets on intended movements. To quantify this, we calculated the intended movement RMSE of the two control algorithms (MKF and CNN) trained on the three different amounts of aggregated training data (one, five, and ten prior datasets).

We found that increasing the number of aggregated training datasets significantly reduced intended movement RMSE (*p* < 0.001, ANCOVA; figure 3(b)). A significant interactive effect was also found between the algorithm and the number of aggregated training datasets, suggesting that the overall reduction in intended movement RMSE from additional aggregated training data was predominantly due to the CNN (*p* < 0.01, ANCOVA). Subsequent pairwise comparisons among the different amounts of aggregated training data showed significant improvement for the CNN (*p* < 0.05), but not for the MKF. Relative to a single training dataset, aggregating five datasets resulted in an 8.8% improvement for the CNN and no improvement for the MKF. This effect was further pronounced with the CNN when aggregating ten datasets. Relative to a single training dataset, aggregating ten datasets resulted in a 24% improvement for the CNN and no improvement for the MKF.

### 3.3. Aggregating prior datasets also improved the robustness of the CNN

**Figure 4.**
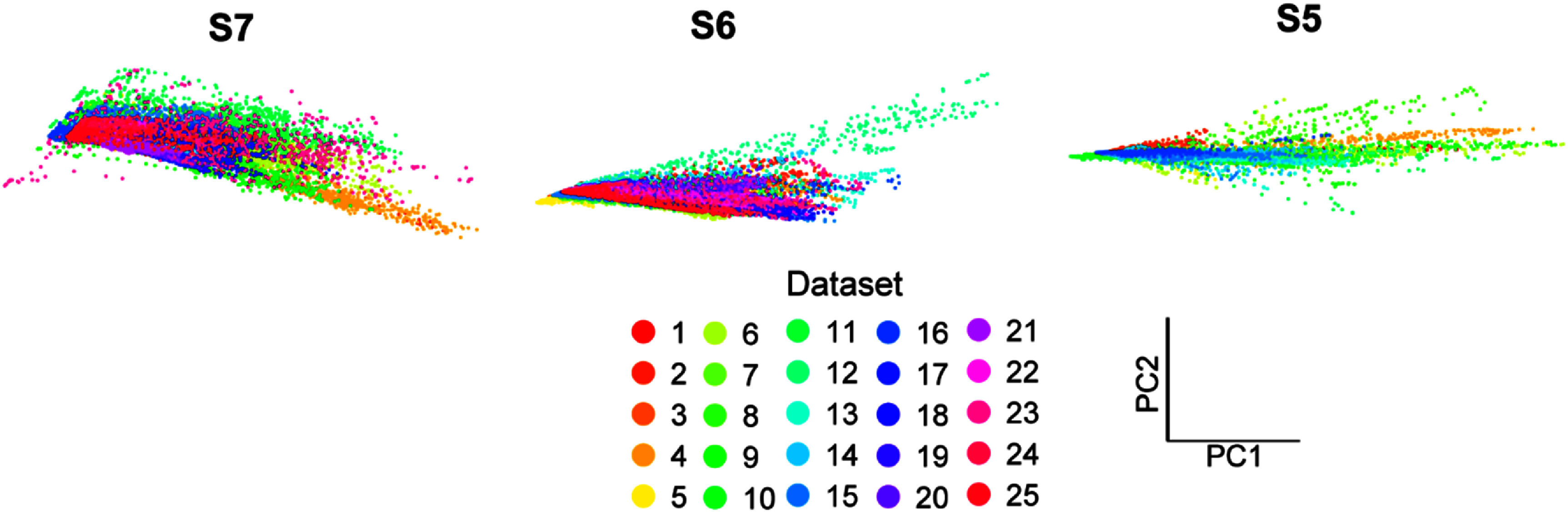
Variance in iEMG feature space across datasets. Principal component analysis was performed across datasets for each patient. Each point represents a time point during the wrist flexion movement within the first two principal components. The large changes in feature space among the different datasets are consistent with the degradation of regression-based algorithm performance over time. Similar changes in feature space among datasets were observed for the other degrees of freedom for each participant (figures S3–S5).

**Figure 5.**
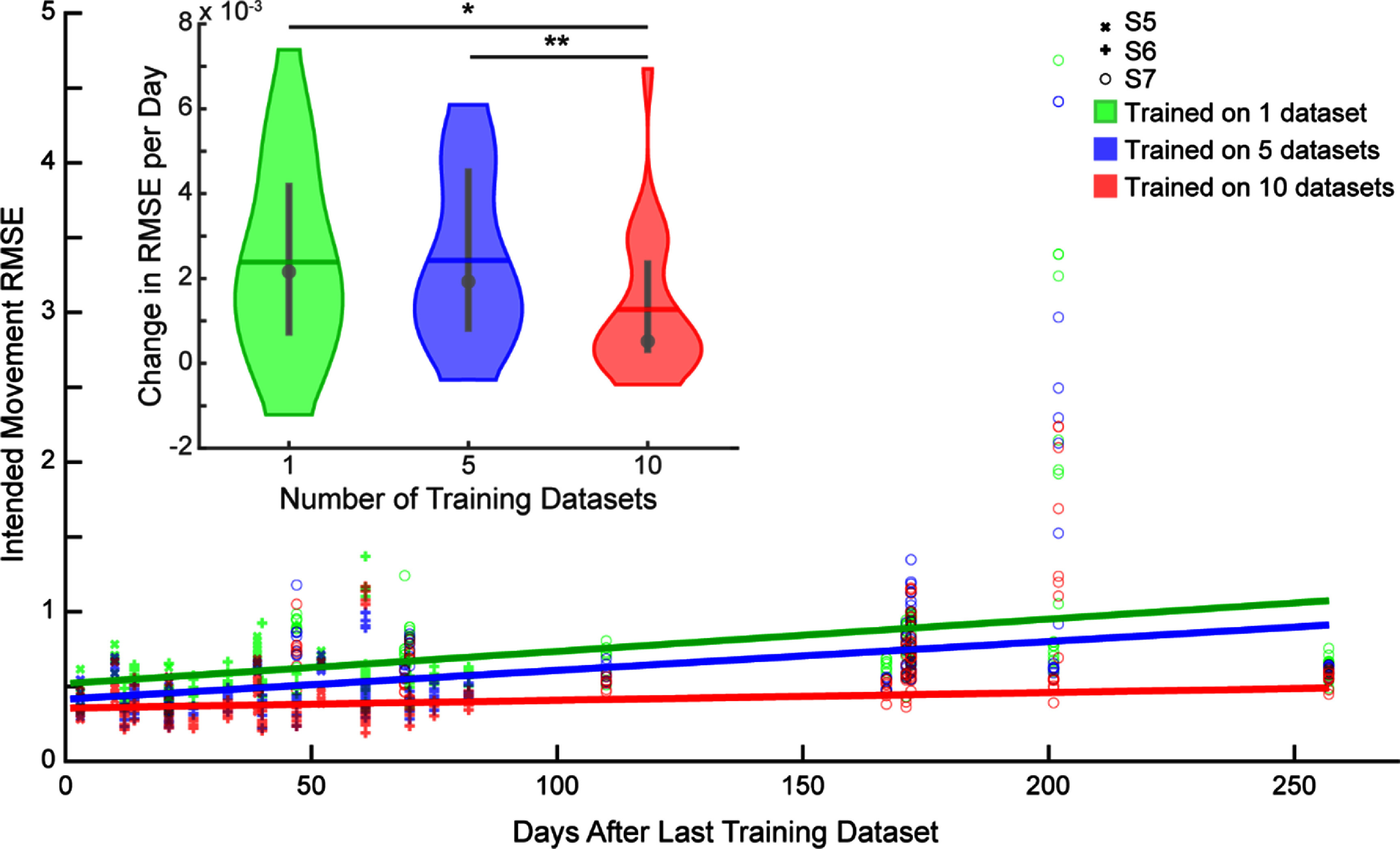
Impact of aggregating training datasets on CNN robustness. Increasing the number of aggregated training datasets resulted in a slower decline in performance over time (i.e. a smaller increase in RMSE/day). The scatter plot shows the RMSE of each of the 8 degrees of freedom for each training dataset. A line of best fit was calculated per participant for each degree of freedom. Lines show the median slope and intercept found from the individually fitted lines. The inset shows the slope of the lines of best fit for the RMSE, as shown in the scatter plot (least square approach). The slope of CNN trained with one dataset was 0.0022 (0.0007–0.0042) RMSE per day, the slope of CNN trained with five datasets was 0.0019 (0.0008–0.0046) RMSE per day, and the slope of CNN trained with ten datasets was 0.0005 (0.0003–0.0024) RMSE per day (median (interquartile range). Violin plots show the kernel density estimation. Colored horizontal lines denote the mean, grey circles denote the median, and grey vertical lines denote the interquartile range. Asterisk (*) denotes *p*< 0.05, and double asterisk (**) denotes *p* < 0.01, Wilcoxon rank sum tests with correction for multiple comparisons. *N* = 24 (three participants, eight DOFs per participant).

The results above demonstrate that aggregating prior datasets can improve algorithm accuracy, particularly for the CNN (figure 3). Next, we explored how aggregating prior datasets impacted the robustness of the algorithm over time. Robustness is particularly important for regression-based control algorithms because small deviations in relative EMG amplitudes lead to errors in both intended and unintended movements. As these errors accumulate over time, performance degrades, and recalibration is required. Indeed, we observed large changes in implanted EMG feature space for each participant (figures 4 and S2–S4) that resulted in worse performance over time (figure 5).

To measure robustness, we quantified the change in overall RMSE for each degree of freedom per participant across all the testing datasets (figure 5). We found a difference in the rates of decay among the different amounts of training data (Kruskal–Wallis, *p*< 0.05). Aggregating five prior datasets had no significant impact relative to a single prior dataset (figure 5-inset). However, aggregating ten prior datasets significantly improved the robustness of the CNN relative to a single prior dataset (*p* < 0.05) and relative to five prior datasets (*p* < 0.01). That is, the CNN trained with ten prior datasets had a slower decline in performance over time (0.0005 (0.0003–0.0024) RMSE per day, 0.0019 (0.0008–0.0046) RMSE per day, and 0.0022 (0.0007–0.0042) RMSE per day for CNNs trained on ten, five, and one datasets respectively, median (interquartile range)).

### 3.4. Baseline performance on real-time TTT

**Figure 6.**
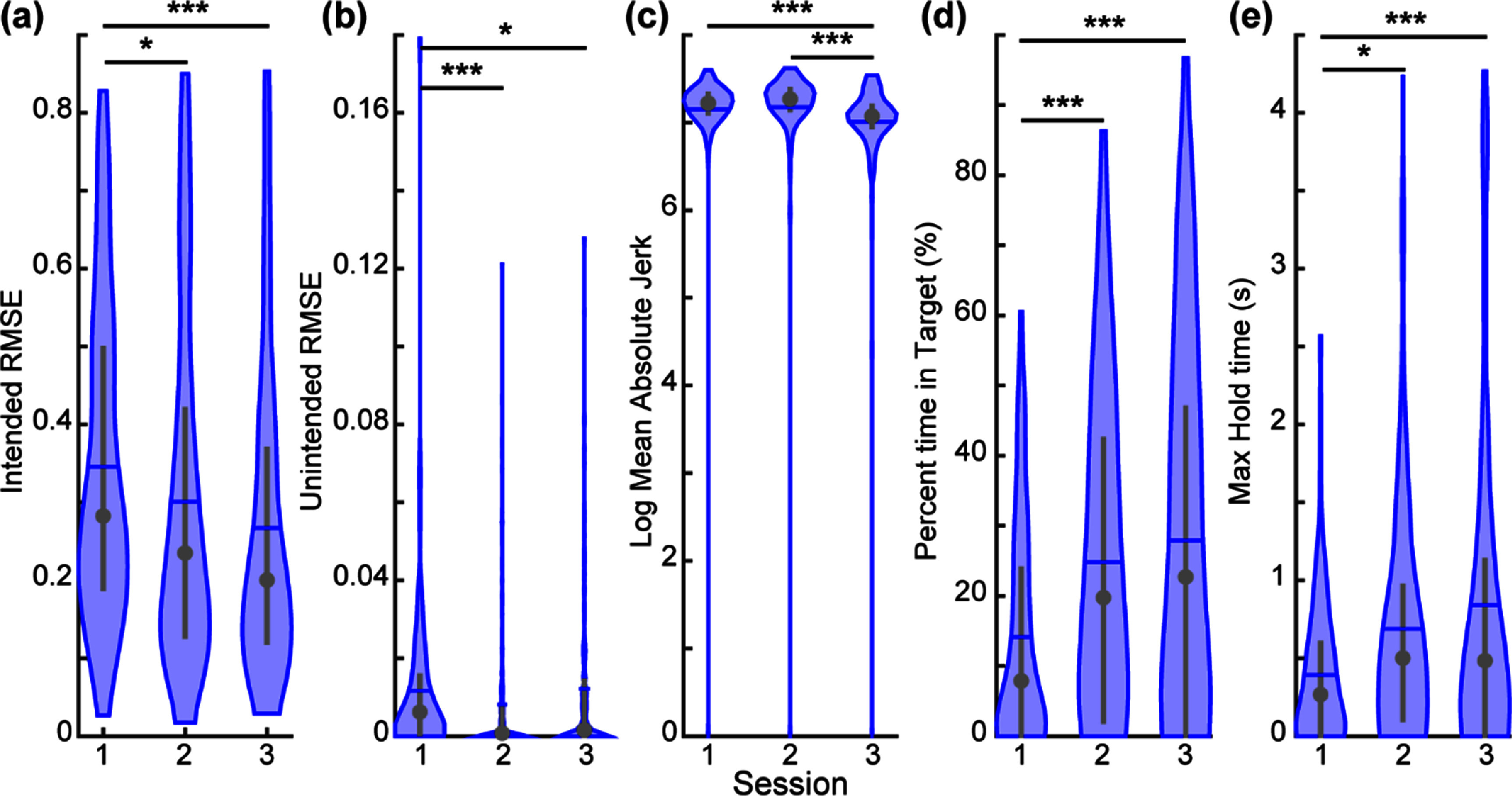
Baseline performance on the real-time target-touching task for S8. The participant completed the target-touching task three times, using a CNN trained on a new dataset collected immediately before the task. As expected, the participant improved on the task over time, and most learning took place relative to session 1. Lower values indicate better performance for intended movement RMSE (a), unintended movement RMSE (b), and log mean absolute jerk (c). Higher values indicate better performance for the percent time in target (d) and max hold time (e). Violin plots show the kernel density estimation. Coloured horizontal lines denote the mean, grey circles denote the median, and grey vertical lines denote the interquartile range. Asterisk (*) denotes *p* < 0.05, double asterisk (**) denotes *p* < 0.01, triple asterisk (***) denotes *p* < 0.001, pairwise comparisons with correction for multiple comparisons. *N* = 120 trials for session 1, and *N* = 240 trials in sessions 2 and 3. Boxplot representations in figure S6.

After demonstrating that aggregating past datasets could improve algorithm performance offline, we next attempted to replicate this online with a transradial amputee actively controlling a virtual prosthesis in real time. To do this, we had the participant complete three training sessions, over the course of two months. Immediately after each training session, the participant also completed a TTT in which they actively attempted to move select degrees of freedom in isolation with proportionality. As expected, the participant showed a modest improvement on the task over the three sessions, presumably due to task familiarity and motor learning (figure 6). These data serve as a performance baseline when using a CNN trained on a single dataset collected immediately before use.

### 3.5. Increasing the number of aggregated training datasets improved online performance

As noted above, the participant completed three training sessions. Immediately after each training session, the participant completed the TTT with a CNN trained on a new dataset collected immediately before. After the second and third training sessions, we also had the participant complete the TTT using a CNN trained on an aggregated dataset consisting of the dataset collected immediately before the task and the dataset(s) collected in the previous training session(s). Thus, the aggregated dataset included more data but also introduced more variability due to changes in EMG recordings over time.

**Figure 7.**
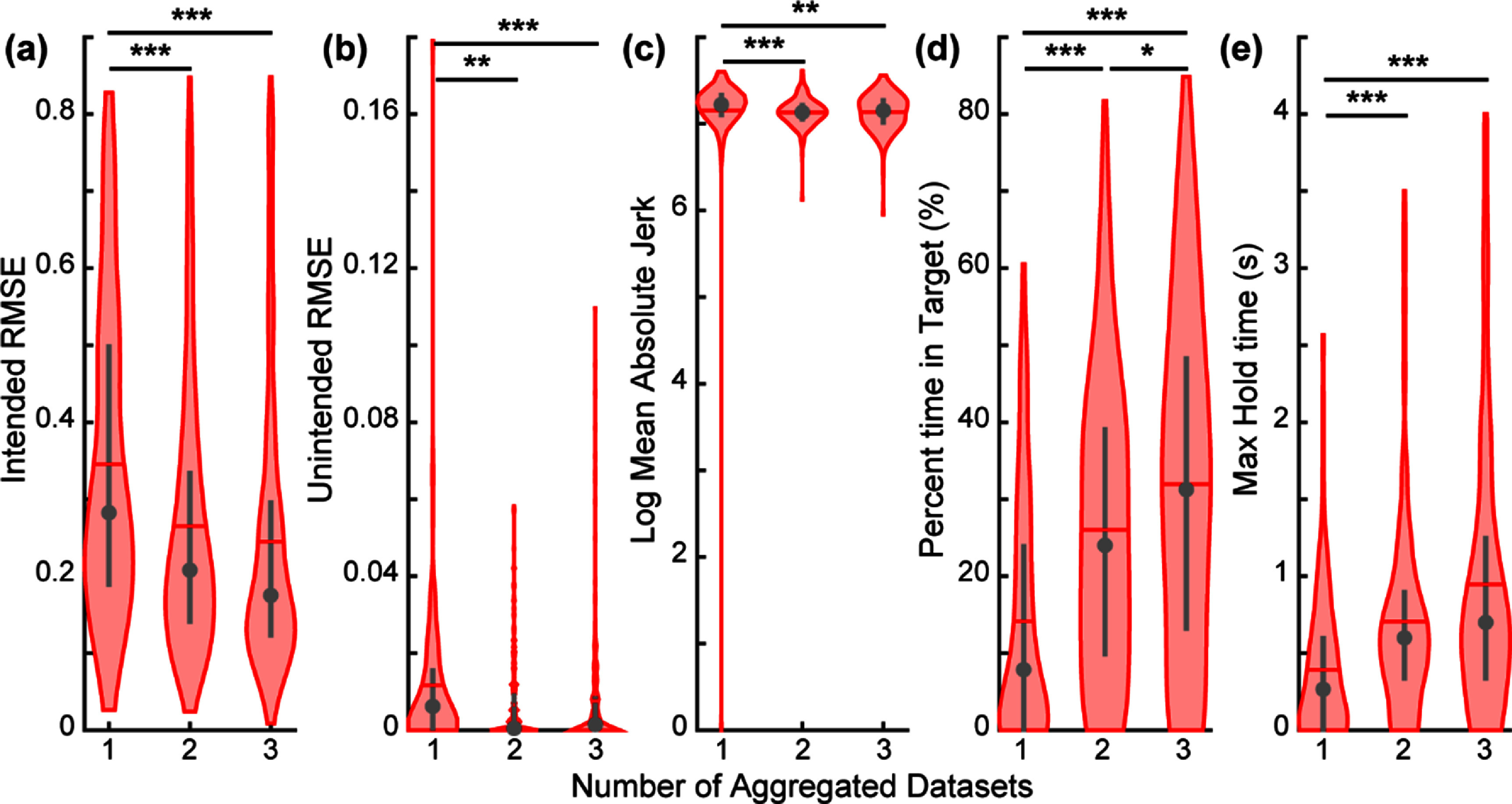
Aggregating past datasets improves performance on a real-time target-touching task. The participant completed the target-touching task three times, using a CNN trained on data aggregated from 1, 2, or 3 past training datasets. Consistent with offline results, increasing the number of aggregated training datasets improved performance. Lower values indicate better performance for intended movement RMSE (a), unintended movement RMSE (b), and log mean absolute jerk (c). Higher values indicate better performance for the percent time in target (d) and max hold time (e). Violin plots show the kernel density estimation. Coloured horizontal lines denote the mean, grey circles denote the median, and grey vertical lines denote the interquartile range. Asterisk (*) denotes *p* < 0.05, double asterisk (**) denotes *p* < 0.01, triple asterisk (***) denotes *p* < 0.001, pairwise comparisons with correction for multiple comparisons. *N* = 120 trials for session 1, and *N* = 240 trials in sessions 2 and 3. Boxplot representations in figure S7.

Consistent with our offline analyses, increasing the number of aggregated training datasets improved task performance (figure 7). This raised the question of whether the gains from aggregated training datasets would outweigh the simple gains associated with human learning (e.g. motor learning, task familiarity).

### 3.6. Data aggregation outperformed data recalibration

We next sought to explicitly answer whether a CNN trained on aggregated past training datasets would outperform a CNN trained on exclusively the most up-to-date training data from immediately before the task. To do this, after the second and third training sessions, we had the participant complete the virtual TTT with both training conditions (same day vs aggregated) using a blinded cross-over design.

**Figure 8.**
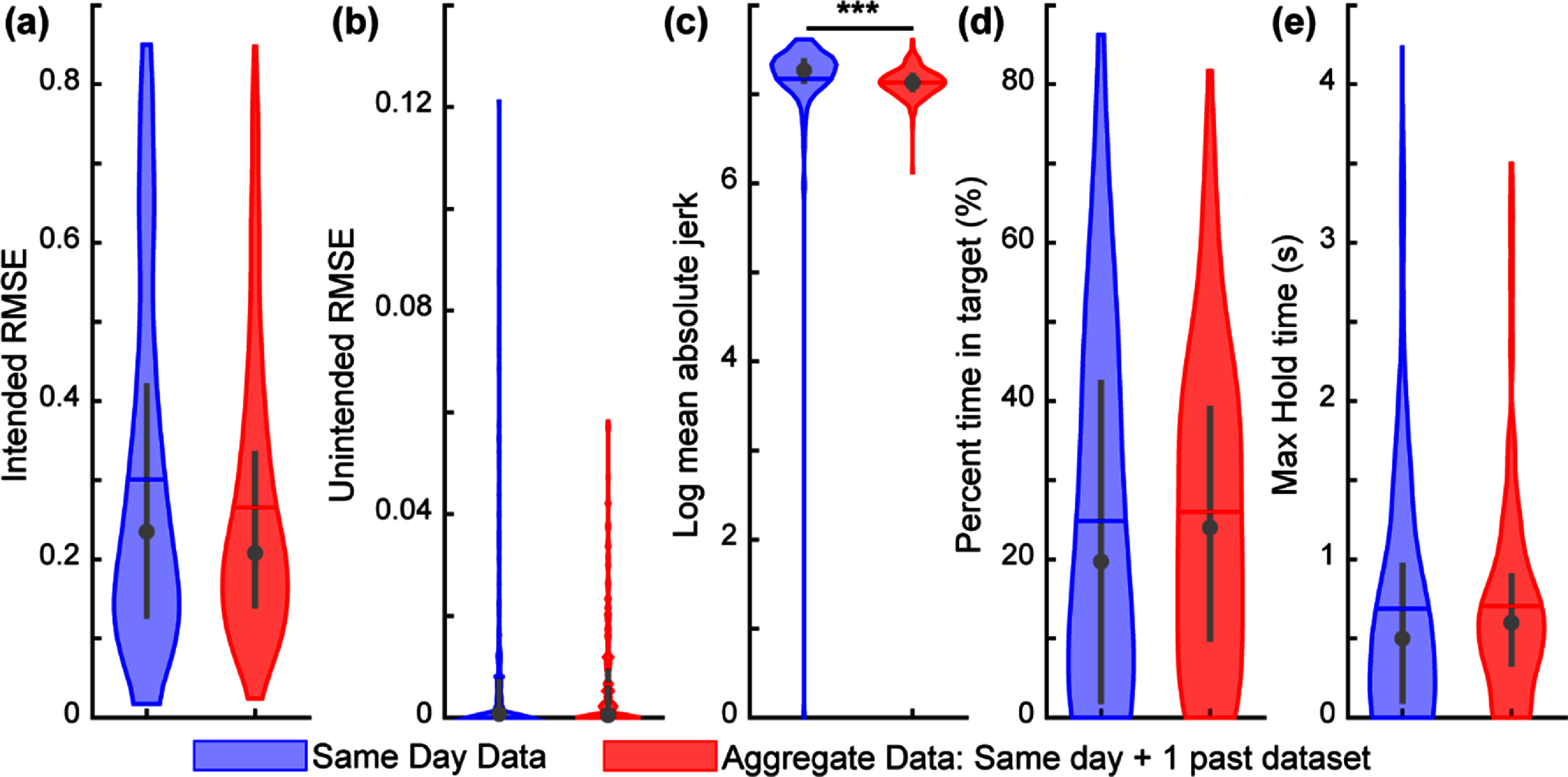
Aggregating a single past dataset slightly improved performance on a real-time target-touching task. Using a blinded cross-over design, the participant completed the virtual target-touching task with both training conditions (same-day data and aggregated data). Aggregated data demonstrated smoother control, as indicated by a lower log mean absolute jerk. Percent time in target and maximum hold time followed a similar but non-significant trend (*p* = 0.15 and *p* = 0.16, respectively). Lower values indicate better performance for intended movement RMSE (a), unintended movement RMSE (b), and log mean absolute jerk (c). Higher values indicate better performance for the percent time in target (d) and max hold time (e). Violin plots show the kernel density estimation. Coloured horizontal lines denote the mean, grey circles denote the median, and grey vertical lines denote the interquartile range. Asterisk (*) denotes *p* < 0.05, double asterisk (**) denotes *p* < 0.01, triple asterisk (***) denotes *p* < 0.001, pairwise comparisons with correction for multiple comparisons. *N* = 240 trials. The data shown here are consistent with those shown in session 2 in figures 6 and 7 and are simply reorganized here for clearer comparison. Boxplot representations in figure S8.

Interestingly, after just the second training session, we observed slight improvements in task performance using the aggregated training datasets relative to exclusively the same-day dataset (figure 8). Specifically, the participant demonstrated smoother control, as indicated by a lower log mean absolute jerk (*p*< 0.001, Wilcoxon rank-sum test; figure 8(c)). Other metrics, such as the percent time in target (figure 8(d)) and maximum hold time (figure 8(e)) followed a similar, but non-significant trend (*p* = 0.15 and *p* = 0.16, respectively).

**Figure 9.**
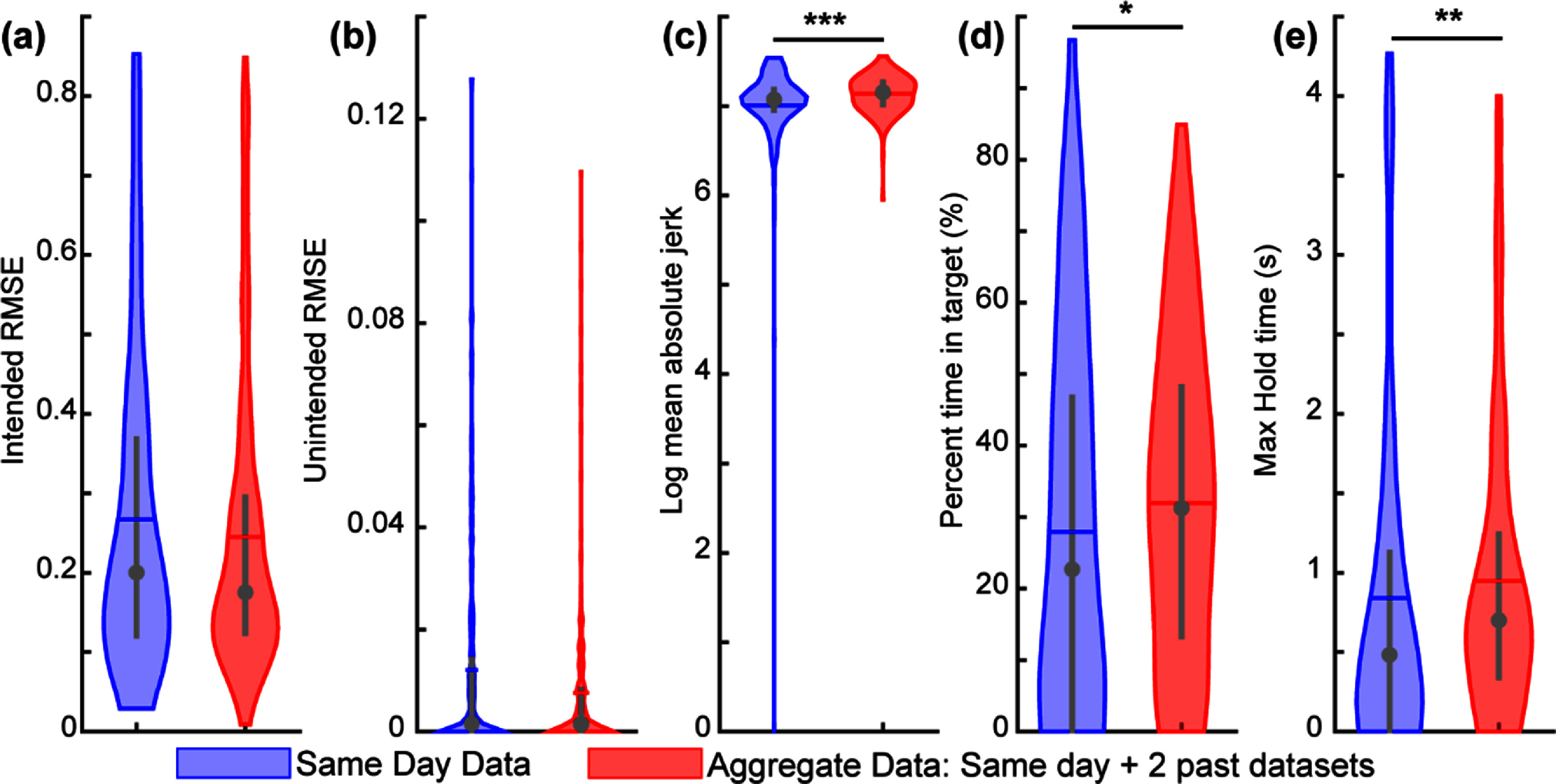
Aggregating two past datasets significantly improved performance on a real-time target-touching task. The participant completed the virtual target-touching task with both training conditions (same-day data vs aggregated data) using a blinded cross-over design. Aggregated data demonstrated better task performance (percent time in target) and significantly better functional control (max hold time), while same-day data demonstrated significantly smoother control (log mean absolute jerk). Lower values indicate better performance for intended movement RMSE (a), unintended movement RMSE (b), and log mean absolute jerk (c). Higher values indicate better performance for the percent time in target (d) and max hold time (e). Violin plots show the kernel density estimation. Coloured horizontal lines denote the mean, grey circles denote the median, and grey vertical lines denote the interquartile range. Asterisk (*) denotes *p* < 0.05, double asterisk (**) denotes *p* < 0.01, triple asterisk (***) denotes *p* < 0.001, pairwise comparisons with correction for multiple comparisons. *N* = 240 trials. The data shown here are consistent with those shown in session 3 in figures 6 and 7 and are simply reorganized here for clearer comparison. Boxplot representations in figure S9.

After the third training session, in which the aggregated datasets consisted of the current dataset and two prior datasets, the participant demonstrated significantly better performance with the aggregated training datasets relative to exclusively the same-day dataset (figure 9). The participant had a significantly greater percent time in target (*p* < 0.05, Wilcoxon rank-sum test; figure 9(d)) and maximum hold time (*p* < 0.01, Wilcoxon rank-sum test; figure 9(e)). Thus, aggregating just two prior datasets significantly improved the participants overall task performance and functional prosthetic control, as demonstrated by percent time in target and maximum hold time, respectively. The participant demonstrated smoother control with the same-day trained algorithm over the aggregate data (*p* < 0.001, Wilcoxon rank-sum test; figure 9(c)).

## 4. Discussion

### 4.1. Summary

Neuroprostheses have advanced beyond simple sequential classification of discrete gestures towards simultaneous and proportional position control of multiple degrees of freedom. However, these new regression-based algorithms are inherently less stable than classification approaches, and as such, they are retrained/recalibrated frequently to ensure only the most recent, up-to-date data are reflected in the algorithm. Here, we introduce and validate an alternative paradigm in which the neuroprosthesis is retrained/recalibrated using an aggregated dataset consisting of both the most recent, up-to-date data and all the prior datasets. In both offline and online studies, we show that training a neuroprosthesis with aggregated training data that includes past datasets significantly outperforms training a neuroprosthesis with exclusively the most recent data.

### 4.2. Mechanism

Why does incorporating past datasets improve neuroprosthesis performance, especially given that neural [29, 32–36] and EMG [8, 29, 37–40] recordings change over time and that, by themselves, these changes degrade algorithm performance [25–28]? We propose that at least two mechanisms are at play. First, incorporating past datasets is a simple way to increase the total amount of training data. Increasing the amount of training data has been shown to improve machine learning performance in multiple domains [3, 82–84] and similarly improves neuroprosthesis performance [25, 65, 74, 85]. Second, training on more diverse data is a simple way to increase the robustness of the algorithm. Providing more robust training data has been shown to improve machine learning robustness in multiple domains [86–90] and similarly has been shown to improve neuroprosthesis performance [46, 51, 91, 92]. Notably, both these mechanisms are enhanced when using more complex, non-linear algorithms [93, 94]. This is consistent with the results presented here (figure 3) and in prior work, showing that a non-linear CNN outperforms a linear KF when there is sufficient data [74, 95]. This work differs from most prior work in that we use prior datasets rather than increasing the amount of data used from the same day of testing.

### 4.3. Minimum number of aggregated datasets across recording modalities

Including outdated or invalid data in the training dataset, in principle, degrades algorithm performance. The benefit of including past data over same-day data depends on a certain level of similarity. Although not directly explored, our findings provide insight into what level of similarity must be maintained and if this approach can be generalized to other recording modalities like sEMG or neural recordings.

Implanted iEMGs, such as those used in this study, have improved signal quality and stability compared to traditional sEMG [29, 85]. Indeed, prior work has shown that the same implanted iEMGs used in this study can be used to classify four discrete classes without recalibration for up to 604 d [37]. However, even implanted iEMG signals change over time (figure 4), and this results in worse performance over time when regressing the kinematic position of a high number of degrees of freedom in real time (figure 5). Thus, aggregating past iEMG datasets is not simply a trivial increase in the total amount of training data; rather, aggregating past iEMG datasets reflects a complex addition of diverse prior data. Interestingly, the present work shows that adding data from just two prior datasets (three times the amount of data) is enough to outperform data from exclusively the same day in a real-time TTT.

Given the lower signal quality and stability of sEMG [29, 85], we anticipate more than two prior sEMG datasets will be needed for aggregated sEMG training data to outperform same-day sEMG training data. Nevertheless, prior work with consistently placed sEMG recording sites has shown that incorporating past data can improve algorithm performance [25, 85]. In [85], for example, performance on a similar TTT was significantly improved using aggregate data from ten prior sEMG datasets relative to a single dataset collected immediately before the task. In the case of commercially available sEMG recordings, which likely experience much greater variability over time than [25, 85], we anticipate more than ten prior sEMG datasets will be needed. Given the frequency of user recalibration and cloud storage use in commercial sEMG systems like Coapt [5, 16, 47], training a model with dozens, if not hundreds, of prior datasets should be feasible. Indeed, Coapt has recently begun data aggregation to reduce recalibration frequency for a linear discriminant analysis algorithm [47]. The results presented in the present manuscript suggest that the improvements seen in [47] would likely be enhanced by leveraging a more complex neural network algorithm. A question specific to sEMG would be if one could leverage data across participants, especially with the datasets collected and stored by commercial sEMG systems. Although EMG data from amputees has been shown to be more variable than from intact individuals [96], studies have shown the possibility of developing a cross-user model that would use data from multiple users and potentially lead toward generalized myoelectric control of prostheses [55, 97]. The present manuscript would support that more data and complex algorithms used in cross-user models would be beneficial.

Neuroprostheses are also frequently controlled with neural data from the cortex [9, 10, 98, 99] or peripheral nerves [41, 100–102]. Both cortical recordings [32, 103, 104] and peripheral recordings [29, 34] are relatively stable over multiple years. Thus, it is also likely that aggregating prior datasets would benefit machine learning algorithms based on neural recordings. For both cortical and peripheral interfaces, dozens of datasets already exist [32, 41] that could be used to explore the efficacy of aggregating past datasets offline.

### 4.4. Diminishing returns and practical considerations of dataset aggregation

An important related question is if or when diminishing returns take effect with dataset aggregation. With the data augmentation technique presented in [51], three iterations of their data augmentation algorithm gave the most benefit to their deep learning control algorithms with diminishing returns for further iterations. In the present study, our offline analyses were limited to a maximum of ten prior datasets based on our preliminary work that suggested diminishing returns beyond ten iEMG datasets [77]. Although our online analyses were limited to only three sessions due to patient time constraints, our offline results suggest further gains could be seen with up to at least ten prior datasets compared to one prior dataset. Future work should explore dataset aggregation with additional recording modalities on functional activities of daily living.

The present and preliminary work [77] utilized the same shallow CNN [65]. The greater the algorithm complexity and capacity, the more likely it is to benefit from additional and more complex data [82, 105]. Indeed, in related work with sEMG, using a deeper neural network enabled further performance improvements with up to 20 past sEMG datasets [85]. Thus, from a practical perspective, demonstrating benefits from aggregating dozens or hundreds of prior datasets will likely require more complex algorithms beyond those presented here.

Another practical consideration is if or when the user can simply stop collecting datasets altogether. Although aggregating ten prior datasets improved algorithm robustness (figure 5), the algorithm performance still decayed over time. We showed that incorporating ten prior datasets into the training data reduced algorithm degradation from 0.0022 RMSE/day to 0.0005 RMSE/day. Future work should explore what level of algorithm degradation (i.e. RMSE increase) justifies recalibration based on performance or user preference. Nevertheless, we suspect an increase of 0.0005 RMSE/day would likely not require daily recalibration.

### 4.5. Co-adaptive learning with dataset aggregation

Although users have a desire for less frequent recalibration [47, 106], they also have a desire for more dexterous and intuitive control [107, 108]. Dataset aggregation presents an opportunity to address both challenges. To the first point, the results suggest that after aggregating an initial number of training datasets, the recalibration frequency can be reduced. Specifically, the results show that after aggregating 10 training datasets there is a 77% improvement in robustness. In terms of practical implementation, if a myoelectric prosthesis was recalibrated after 2 h of use, then with dataset aggregation the individual would instead recalibrate after 3.5 h of use. As more and more datasets are aggregated, the recalibration frequency may continue to decrease. To the second point, we propose that, beyond simply reducing recalibration frequency, dataset aggregation presents a unique opportunity to slowly accumulate more complex and diverse training data that yields more dexterous and intuitive neuroprostheses. Under this proposed paradigm, instead of recalibrating with the same subset of gestures every day, the user could first perform a fixed number of calibrations with basic gestures and then progress to increasingly complex gestures and motions while still retaining algorithm performance on past gestures and motions. In other words, the results presented here show that existing models can be trained on diverse data to improve stability and performance. Because the algorithm is more stable, users would not need to recalibrate the same basic gestures each day. Then, to introduce more diversity among the datasets, users could begin training with new task-specific gestures. For example, users could first aggregate multiple datasets consisting of grasping, then aggregate multiple datasets for wrist rotation, and then aggregate datasets involving combination movements like opening a door handle or pouring a glass of water. We speculate that this approach could further improve the performance of regression algorithms, as prior work has shown task-specific training improves performance on activities of daily living [46].

In this light, dataset aggregation can be viewed as a co-adaptive learning approach in which both the human and the machine continue to learn and retain past knowledge over time. Consistent with prior work on co-adaptive neuroprostheses [109, 110], our results show better overall performance with dataset aggregation, where both the human and machine retain past knowledge, relative to same-day data, where only the human retains past knowledge (figures 6–9). Reducing the learning period with prostheses could help increase adoption during the critical golden window after amputation [111].

Although dataset aggregation has the potential to improve robustness and dexterity of myoelectric prostheses, we also recognize that users would still be required to collect multiple datasets before any benefits are seen. Additionally, the larger datasets could slow the calibration process. However, given the potential advantages of leveraging past data, and the rapidly increasing computational power available for cloud and edge computing, these concerns are minimal. Indeed, even commercial sEMG systems are now leveraging dataset aggregation, cloud computing, and computationally efficient models to reduce recalibration frequency [47].

### 4.6. Limitations

Our study design and statistical analyses for the offline analyses of group data do not allow any clear conclusions regarding whether results from data aggregation or the type of algorithm (CNN or MKF) may generalize to the general population. In statistical terms, the group data were collapsed across all the relevant participants (i.e. analysed as if from a single participant), and hence do not allow the necessary estimate of the population. However, informal inspection indicates that the CNN and MKF had an equal number of ‘wins’ for the performance of the MKF vs CNN (figure 3), possibly with the MKF doing relatively better with smaller data sets. From a practical perspective, participants may fare better with the simpler MKF early in prosthetic use but switch to more complex algorithms as available data sets grow.

Unlike offline analyses, online tests allow interactive corrections from the participant and may more accurately reflect real-life performance. Aggregating multiple data sets yielded some statistically significant effects for the one participant tested. However, results were mixed across the five different metrics, and the magnitudes of effects were small. The overall potential clinical benefits may depend on individual participants, tasks, experience, and other parameters.

## 5. Conclusion

This work introduces the concept of dataset aggregation for regression-based prosthetic control, in which past training datasets are aggregated and reused in all future training datasets. We show that dataset aggregation increases prosthetic learning, accuracy, and robustness, particularly for non-linear algorithms. Together, these results support a paradigm shift for the field of neuroprostheses away from daily data recalibration for linear algorithms and towards daily data aggregation for non-linear algorithms.

## Data Availability

The data cannot be made publicly available upon publication because they contain sensitive personal information. The data that support the findings of this study are available upon reasonable request from the authors, pending approval by the University of Utah Institutional Review Board.
